# Prediction of hearing aid cognitive outcomes in age-related hearing loss

**DOI:** 10.3389/fnagi.2025.1548526

**Published:** 2025-02-20

**Authors:** Patrice Voss, Zaida Escila Martinez-Moreno, Francois Prévost, Anthony Zeitouni, Alejandro Lopez Valdes, Etienne de Villers-Sidani

**Affiliations:** ^1^Department of Neurology and Neurosurgery, Montreal Neurological Institute, McGill University, Montreal, QC, Canada; ^2^Département of Audiology and Speech-Language Pathology McGill University Health Center, Montreal, QC, Canada; ^3^Department of Otolaryngology-Head and Neck Surgery, McGill University, Montreal, QC, Canada; ^4^Trinity College Institute of Neuroscience, Trinity College Dublin, Dublin, Ireland; ^5^Global Brain Health Institute, Trinity College Dublin, Dublin, Ireland; ^6^Department of Electronic and Electrical Engineering, School of Engineering, Trinity College Dublin, Dublin, Ireland; ^7^Trinity Center for Biomedical Engineering, Trinity College Dublin, Dublin, Ireland

**Keywords:** hearing aids, cognition, speech-in-noise, hearing loss, aging, outcome measure

## Abstract

Although the phenomena underlying cognitive decline and dementia are complex, there is growing evidence suggesting that degraded sensory inputs caused by age-related hearing loss may play a central role in accelerating cognitive decline in older individuals. Further supporting this notion is evidence that hearing augmentation with hearing aids can mitigate hearing loss-related cognitive impairments. Despite this evidence, few studies have attempted to investigate hearing aid efficacy with a focus on cognitive outcome measures. In this preliminary study, we sought to determine if certain demographic and audiological factors are linked to individual differences regarding observed cognitive changes following hearing aid use. We show that several factors can explain large portions of the variance observed in cognitive score changes following short-term hearing aid use in first-time users, suggesting that it might be possible to develop predictive algorithms to determine individualized estimates of the cognitive benefit of hearing aid use. Future studies with larger sample sizes are warranted, in particular, to explore a wider array of cognitive functions, investigate a greater range of potential predictors, and better quantify their relative contribution to outcome measure estimates.

## Introduction

Although the exact causes of many dementias and age-related cognitive impairments remain unknown, growing evidence suggests that age-related hearing loss could play a central role in their development (Meister et al., 2015; Deal et al., 2017; Lin et al., 2011, 2013; Curhan et al., 2019; Humes, 2021; Chern et al., 2022). Large cohort studies have provided substantial evidence that hearing impairment in older adults is independently associated with accelerated cognitive decline and incident dementia, with some indicating that for every 10 decibels in hearing loss, cognitive impairment significantly increases (Golub et al., 2020) and there is a substantial increase in the risk of developing dementia (Loughrey et al., 2018).

Additional support comes from a Lancet Commission article, which found that ARHL was responsible for 7% of the risk of developing dementia, making ARHL the potentially modifiable risk factor with the highest risk of nine identified factors associated with dementia (Livingston et al., 2024). A biological gradient (e.g., dose-response) was also identified whereby the risk ratio of dementia is increased as a function of the magnitude of the hearing loss. Finally, a recent umbrella review (a systematic review of multiple systematic reviews) concluded that ARHL is significantly associated with cognitive impairment and dementia and may be an important risk factor for both (Ying et al., 2023).

The link between hearing loss and cognition is further supported by several studies investigating the effect of hearing aid use on cognitive abilities. Initial cross-sectional studies showing that hearing use attenuates cognitive decline further suggested that ARHL may play a causal role in the development of cognitive impairments (Amieva et al., 2015; Dawes et al., 2015; Castiglione et al., 2016; Qian et al., 2016; Ray et al., 2018; Grenier et al., 2024). More recently, longitudinal studies have demonstrated reduced cognitive decline or cognitive gains following prolonged use of hearing aids (Maharani et al., 2018; Sarant et al., 2020, 2024; Cominetti et al., 2023; Glick and Sharma, 2020). Taken together, these findings strongly support the use of hearing aids as an important tool in the fight against cognitive decline and dementia—in fact, some authors have argued we should provide hearing aids much earlier in the course of hearing loss and promote their use more aggressively (Roalf and Moberg, 2016).

What is unclear from the literature on the cognitive benefit of hearing aid use is to what extent the effect is widespread and what role is played by individual differences – although one recent study of hearing aid use showed that hearing intervention may reduce cognitive change in older adults at increased risk for cognitive decline but not in populations at decreased risk for cognitive decline (Lin et al., 2023). It is also unclear whether certain predictors of the cognitive benefit of hearing aid use can be identified. To our knowledge, most studies investigating the predictors of hearing aid success have focused on auditory and general satisfaction outcome measures, not cognitive ability. More specifically, investigated outcomes typically include either speech intelligibility (Lopez-Poveda et al., 2017) or patient-reported outcome measures as typically assessed via the International Outcome Inventory for Hearing Aids (IOI-HA; Houmøller et al., 2022; Jang et al., 2024; Wu et al., 2019; Lansbergen et al., 2023).

Given the growing body of research supporting the clinical use of hearing aids for the prevention of cognitive decline, we sought to investigate the possibility of identifying patients for whom hearing aids could be particularly beneficial from a cognitive benefit perspective. In this preliminary exploratory study, we attempt to identify predictors of hearing aid outcomes in first-time hearing aid users, as measured by improvements in standard cognitive tests following short-term hearing aid use.

## Methods

### Participants

Fourteen older adults [5 females; age = 77.2 years (SD = 6.1)] with ARHL participated in the study. All audiological measures were obtained by a licensed audiologist. Hearing loss inclusion criteria consisted of an average pure-tone threshold exceeding 35 dB of normal hearing for frequencies between 0.25 and 8 kHz, with a max slope of 20 dB/octave between 1 and 4 kHz. Hearing loss exclusion criteria consisted of a reverse slope (max −5dB/octave) between 0.25 and 1 kHz, an asymmetrical hearing loss (max 10 dB average difference between ears), and hearing loss related to noise-induced occupational hearing loss or tinnitus. No participant had a diagnosed major neurocognitive disorder at the time of examination, and all were further screened with the Montreal Cognitive Assessment (MoCA) to ensure that none fell below the cutoff score outlined in the updated criteria by Carson et al. (2018)—all scores were ≥24. Participants also had no history of neurological or psychiatric conditions and had never worn hearing aids. All study procedures were approved by the Neurosciences Panel of the MUHC Research Ethics Board and all subjects provided written informed consent.

### Study design and hearing aid fitting

After having undergone audiological examination and consented to take part in the study, participants were fitted with bilateral Oticon hearing aids (Oticon Inc., Somerset, New Jersey, USA) by a licensed audiologist and were instructed to wear them for at least 8 h per day for 3 months. Hearing aids were programmed to match individual participant audiograms and had proprietary noise-reduction functions activated. All study data was collected during two testing sessions, 12 weeks apart. Participants wore the hearing aids for 7–10 days to allow time for acclimatization with the devices before taking part in the first testing session when they underwent speech-in-noise testing and several neuropsychological tests. Participants underwent the same tests within 7–10 days following the 12-week period of hearing aid use.

### Speech-in-noise (SIN) testing

SIN perception ability was evaluated with a hearing-in-noise task (HINT; Nilsson et al., 1994). Participants were asked to repeat 20 short sentences embedded in multispeaker babble noise created from four speakers (two female), which were presented in either French (Vaillancourt et al., 2005) or English (Nilsson et al., 1994) depending on the subject's native language. The signal-to-noise (SNR) varied from trial to trial following a staircase paradigm—the sound level of each sentence is adjusted (relative to the multi-speaker babble) based on the subject's response to the previous sentence. Performance was scored on a word-by-word basis and the staircase procedure was designed to establish an individual signal-to-noise (SNR) hearing threshold to achieve a 50% success rate for correctly repeated words over the entire 20-sentence run.

### Neuropsychological testing

Participants completed six neuropsychological that were selected for their good reliability and validity in measuring a varied array of cognition functions known to be affected in aging (Park et al., 2000; Faria et al., 2015): (1) Rey Auditory Verbal Learning Test (RAVLT; Rey, 1941) to assess verbal learning, (2) Aggie Figures Learning Test (AFLT; Jones-Gotman, 1977) to serve as a visual analog to RAVLT, (3) the Wechsler Adult Intelligence Scale-Revised (WAIS-IV; Wechsler, 2008) Digit Symbol-Coding (CD) and Symbol Search (SS) tests to obtain a measure of processing speed; executive functions were evaluated with the Delis-Kaplan Executive Function System (D-KEFS; Delis and Kaplan, 2001) subtests, (4) Verbal Fluency Test, and (5) Trail Making Tests parts I and II, and 6) The Tower of London test (Shallice, 1982). Alternate forms were used for the RAVLT and AFLT to mitigate potential learning effects.

### Outcome measures for data analysis

Given the number of tests used and the multiple possible outcome measures for each, we selected, *a priori*, one outcome measure per test for use in subsequent analyses. For the RAVLT and AFLT, the total number of properly recalled items across the five trials was selected, whereas the processing speed Index (PSI) was selected for the Digit Symbol-Coding and Symbol Search tests of the WAIS. Regarding the executive function measures, the number-letter switching time (D-KEFS trail test), the total number of produced words (D-KEFS fluency test), and the number of problems solved (Tower of London) were selected. The selected HINT outcome measure was the 50%-correct SNR threshold.

The following demographic and audiological measures were selected as potential predictors of cognitive outcomes: age, sex, education, average low-frequency (250, 500, 1,000, and 2,000 Hz) hearing threshold (***LF Threshold***), average high-frequency (4,000, 6,000, and 8,000 Hz) hearing threshold (***HF Threshold***), audiogram ***Threshold Slope*** (from 250 to 8,000 Hz), and the baseline 50%-correct SNR threshold (***HINT SNR Threshold***). Baseline and follow-up measurements were compared with paired *t*-tests, whereas the relationships between outcome measures and predictors were investigated with Pearson correlation coefficients and linear regression models. Due to the preliminary nature of the present study and the small sample size, we opted not to use statistical corrections for multiple comparisons to avoid ruling out potential leads for future research.

## Results

Following the 12-week hearing aid-wearing period, participants showed score improvements in all cognitive test outcome measures except for the verbal fluency measure (see Table 1—note that the +/− symbols were used to reflect improvement/worsening on a given test outcome measure, and not a score increase/decrease). Of those measures that improved, only three improvements were statistically significant (*p* ≤ 0.05): the PSI (symbol search and coding), the trail number-letter sequence time, and the number of correctly recalled items in the AFLT.

**Table 1:** Score change between the baseline and the 12-week follow-up for the six cognitive tasks and the SIN SNR threshold.

	**RAVLT**	**AFLT**	**WAIS CD-SS**	**D-KEFS trails**	**D-KEFS fluency**	**Tower of London**	**HINT-SNR**
**Score Change**	+7.6%	**+21.2%**	**+7.2%**	**+16%**	−4.2%	+24%	**+28%**
***p*** **-value (** ***t*** **-test)**	0.139	**0.014**	**0.014**	**0.05**	0.286	0.15	0.21
**Effect size**	0.422	**0.792**	**0.757**	**0.569**	0.297	0.409	0.231

Significant (*p* < 0.05) changes are bolded and with gray-shaded cells. Note that for trails test, the sign (direction) of the score change was flipped so that an increased value (“+”) indicates improvement. RAVLT: Auditory Verbal Learning Test (total correct recalls after 5 trials); AFLT: Aggie Figures Learning Test (total correct recalls after 5 trials); WAIS CD-SS: Wechsler Adult Intelligence Scale-Revised Digit Symbol-Coding (CD) and Symbol Search (SS; processing speed index); D-KEFS Trails: Delis-Kaplan Executive Function System Trails 1 and 2 (number-letter switching time); D-KEFS Fluency: Delis-Kaplan Executive Function System Verbal Fluency Test (total number of words produced); Tower of London Test (number of problems solved); HINT-SNR signal-to-noise ratio of the speech perception threshold.

Although the HINT SNR threshold was reduced (improved SIN perception) on average by a large percentage, the improvement was not statistically significant due to important variability across the subjects (six subjects had slightly higher thresholds following the interventional period).

**Figure 1:**
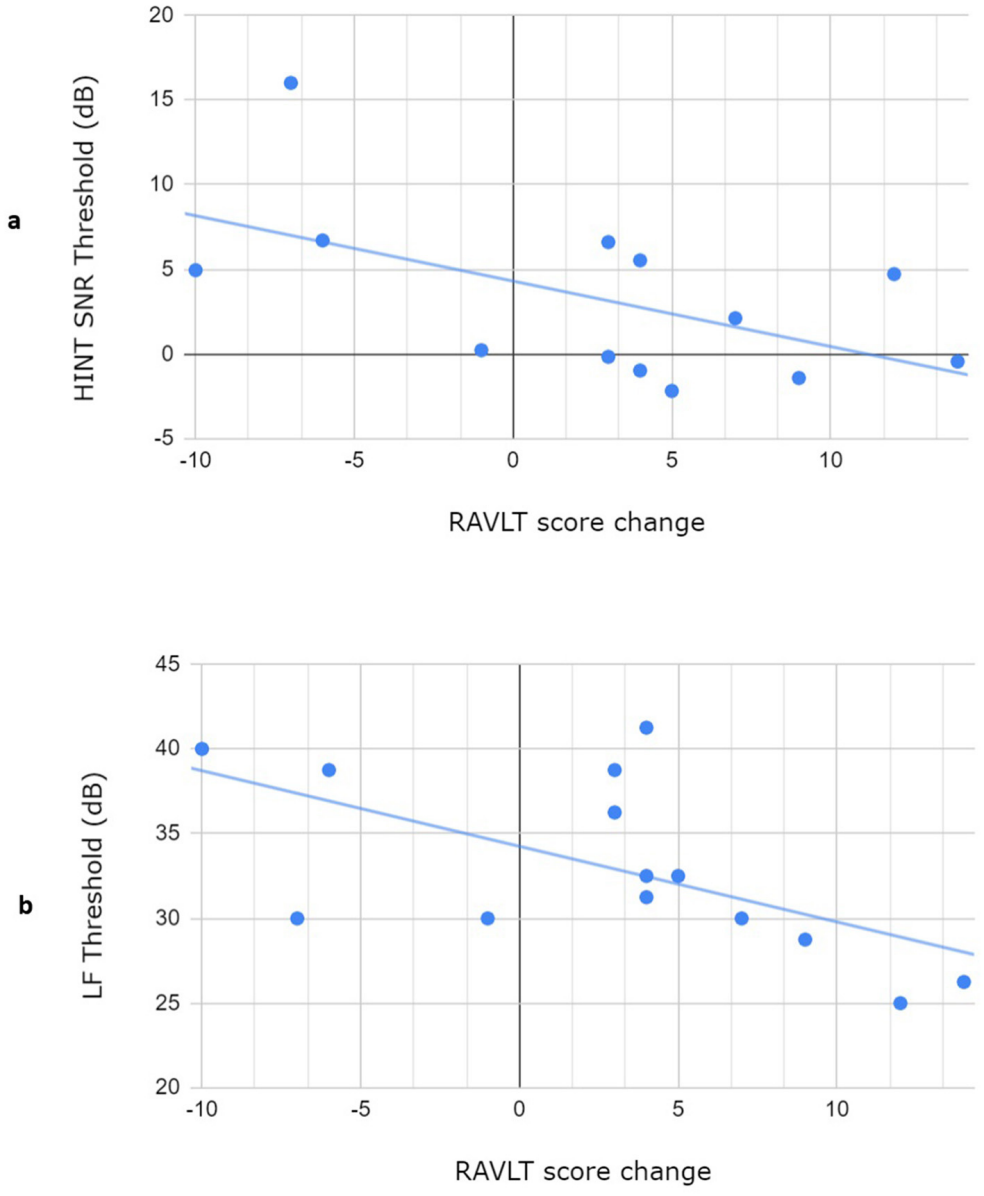
Scatterplots depicting the three statistically significant correlations: **(A)** between the HINT SNR threshold measured at baseline and the change in RAVLT score and **(B)** between the LF threshold measured at baseline and the change in RAVLT score. LF Threshold: hearing average threshold of the low-frequency sounds used in the audiometric assessment; HINT SNR Threshold: HINT tasks signal-to-noise ratio of the speech perception threshold; RAVLT: Auditory Verbal Learning Test (total correct recalls after 5 trials).

As a first step to investigate the relationship between predictors (baseline demographic and audiological factors) and outcome measures, we computed the Pearson correlation coefficients between the predictors and the cognitive test score changes and report the results in Table 2. Given the small sample size, we report not only statistically significant correlations but also correlation coefficients ≥0.3 or ≤ -0.3, values selected to include all linear relationships that are considered at least of moderate strength (Ratner, 2009). Scatterplots in Figure 1 depict the two statistically significant correlations between predictors (LF threshold and HINT-SNR) and the change in RAVLT score. The RAVLT and AFLT were the two cognitive outcomes for which the score change had the largest number of moderate correlations with predictors (including all r ≥0.3). There was a marked drop in the number of predictors that correlated moderately with the score change in the other cognitive tests, with the WAIS Symbol Search and Digit Symbol-Coding and Tower of London score changes only correlating with one predictor. No moderate correlations were found for the D-KEFS Trails test.

**Table 2:** Correlation matrix between demographic/hearing baseline values and the change in score in the six cognitive tests.

	**RAVLT**	**AFLT**	**WAIS symbol search**	**D-KEFS trails**	**D-KEFS fluency**	**Tower of London**
Age	−0.44		−0.46			
Sex	−0.33					0.43
Education		0.5				
Threshold slope		0.4			−0.34	
LF threshold	**−0.6**				**−0.57**	
HF threshold	−0.3	−0.39				
HINT-SNR	**−0.53**	−0.31				

Only correlation coefficients ≥ |0.3| are indicated, and significant (*p* < 0.05) correlations are bolded with gray-shaded cells. RAVLT: Auditory Verbal Learning Test (total correct recalls after 5 trials); AFLT: Aggie Figures Learning Test (total correct recalls after 5 trials); WAIS CD-SS, Wechsler Adult Intelligence Scale-Revised Digit Symbol-Coding (CD) and Symbol Search (SS; processing speed index); D-KEFS Trails: Delis-Kaplan Executive Function System Trails 1 and 2 (number-letter switching time); D-KEFS Fluency: Delis-Kaplan Executive Function System Verbal Fluency Test (total number of words produced); Tower of London Test (number of problems solved); HINT SNR Threshold: HINT tasks signal-to-noise ratio of the speech perception threshold; LF Threshold: hearing average threshold of the low-frequency tones used in the audiometric assessment; HF Threshold: hearing average threshold of the high-frequency tones used in the audiometric assessment.

Finally, we explored the relationship between the predictors and each cognitive change score via linear regression models. Only predictors identified in Table 2 (with correlation coefficients that were ≥ 0.3 for a given cognitive outcome measure) were used as potential independent variables for each outcome measure regression model. The best model for the RAVLT score change had an adjusted R-square of 0.482 (*r* = 0.775, *r*^2^ = 0.601, *p* = 0.02) and included three predictors (low-frequency hearing threshold, HINT SNR threshold, sex). In contrast, the best model for the AFLT had an adjusted R-square of 0.463 (*r* = 0.766, *r*^2^ = 0.587, *p* = 0.03) and included three features (education, hearing threshold slope, and HINT SNR threshold). No model tested for the other cognitive outcome measures produced had an adjusted r-square greater than that obtained with the best correlation with a single predictor.

## Discussion

In light of the growing body of evidence demonstrating that hearing aids can mitigate hearing loss-related cognitive impairments, we sought to investigate whether individual demographic and audiological factors could contribute to predicting outcome measures of cognitive benefit in first-time hearing aid users. Our main objective was not to quantify specific predictors or specifically quantity their predictive power for cognitive outcomes, but rather, given the small sample and preliminary nature of the present study, demonstrate that it is possible to identify such predictors to pave the way for follow-up investigations that could lead to the development of algorithms that identify individuals at risk of cognitive decline who might most benefit from hearing aid use. Our findings indicate that several demographic (sex, age, education) and audiological (characteristics of the audiogram, speech-in-noise comprehension) factors are moderately-to-strongly correlated with changes in cognitive test scores following short-term hearing aid use (12 weeks).

The present study was not designed nor powered to make specific claims regarding what cognitive domains most benefit from hearing aid use. However, our findings are mostly in line with those of Glick and Sharma (2020), who found significant improvement in processing speed, visual working memory and executive functions scores following 6 months of hearing aid use, but without a significant improvement in auditory working memory. Why this would be the case is unclear at the moment, but our findings not only indicate large variability with regards to the score change observed in our auditory working memory task but also that this variability appears to be tightly related to demographic and audiological factors. Indeed, using only three parameters as predictors (low-frequency hearing threshold, HINT SNR threshold, and sex), a linear regression model was able to explain 48% of the variance observed in the RAVLT score difference between baseline and follow-up. These findings are also in line with previous research showing that audiometric hearing impairment predicted short-term cognitive declines in auditory verbal learning tasks (Armstrong et al., 2020). The finding that sex is a potentially important predictor in this instance is in line with well-known sex differences in auditory-verbal memory in educated older adults (Gale et al., 2007; McCarrey et al., 2016). The finding that the HINT SNR threshold was inversely correlated with improvement in the verbal memory task, however, was less expected. This would seem to indicate that poor baseline SIN perception limits the ability of HAs to improve auditory verbal memory following short-term use. This conclusion might also generalize to other cognitive domains, as the HINT SNR threshold was also negatively correlated to improvement in the visual memory task (although the correlation was not statistically significant).

Direct comparisons with other studies showing the beneficial effects of hearing aids on cognition are more difficult either because the cognitive domains studied differed (e.g., attention, learning, global working memory) or focused solely on a global cognitive function score as the outcome measure of interest, such as the MMSE (Sarant et al., 2020, 2024; Cominetti et al., 2023). Nonetheless, our findings also further advocate that standard audiological screening procedures could benefit from the inclusion of speech-in-noise perception tests in addition to the standard audiometric assessment. In line with our findings, recent research has linked speech-in-noise comprehension (if not more so than standard audiometric thresholds) to cognitive decline (Arjmandi et al., 2024; Nemati et al., 2024).

The present preliminary study is not without limitations. First and foremost is the small sample size, which resulted in a small number of significant correlation coefficients despite several linear relationships that could be qualified as moderate or greater. Furthermore, the small sample limits the generalizability of several of the fundings, most notably regarding the specific factors that were identified as predictors of the selected cognitive measures of interest. The study sample might also have been biased in that all participants were willing to actively take measures to improve the hearing and participate in this intervention study. Other limitations include outcome measures that didn't cover the full spectrum of cognitive domains and the use of only a select few demographic and audiological predictor variables. The inclusion of additional relevant variables will no doubt improve the precision of predictive models aimed at identifying the parameters that best predict cognitive benefit. Finally, although selected as such due to the preliminary nature of this study, the short duration of the hearing aid augmentation period most likely doesn't provide the full extent of the effects of prolonged hearing augmentation via hearing aids.

Despite its limitations, we believe the present preliminary study sheds an important light on an under-investigated aspect of hearing augmentation and its potential to mitigate or offset the effects of age-related cognitive decline. Although there is increasing evidence supporting the beneficial effects of hearing aid use on cognition, little is known about what contributes to successful cognitive outcomes and if such outcomes can be predicted prior to hearing aid use. As highlighted earlier, better-powered studies are warranted to further our understanding of the relationship between individual predictors and cognitive outcomes to eventually develop strong predictive algorithms, which in turn could be used to help select patients for whom hearing aids could prescribed as a means to mitigate age-related cognitive impairments.

## Data Availability

The raw data supporting the conclusions of this article will be made available by the authors, without undue reservation.
